# *Beta vulgaris* L.—A Source with a Great Potential in the Extraction of Natural Dyes Intended for the Sustainable Dyeing of Wool

**DOI:** 10.3390/plants12101933

**Published:** 2023-05-09

**Authors:** Vasilica Popescu, Alexandra Cristina Blaga, Dan Cașcaval, Andrei Popescu

**Affiliations:** 1 Department of Chemical Engineering in Textiles and Leather, Faculty of Industrial Design and Business Management, “Gheorghe Asachi” Technical University of Iasi, 700050 Iasi, Romania; 2 Department of Organic, Biochemical and Food Engineering, “Cristofor Simionescu” Faculty of Chemical Engineering and Environmental Protection, “Gheorghe Asachi” Technical University of Iasi, 700050 Iasi, Romania; alexandra-cristina.blaga@academic.tuiasi.ro (A.C.B.); dan.cascaval@academic.tuiasi.ro (D.C.); 3 Department of Mechanical Engineering, Mechatronics and Robotics, Faculty of Mechanical Engineering, “Gheorghe Asachi” Technical University of Iasi, 700050 Iasi, Romania; andrei.popescu@academic.tuiasi.ro

**Keywords:** betalains, deprotonation, decarboxylation, dyeing, pectinase, pressure, stabilizer

## Abstract

*Beta vulgaris* L. is a biennial plant easily accessible all over the world, rich in various biologically active compounds, especially a class of extremely bioactive pigments known as betalains. These dyes predominate in the pulp and peels of beetroot, which is why they can be valorized in food, medicine or in the textile industry. In this work, betalains extractions were carried out applying 3 sustainable options: (1) dissolving/solubilizing betalains in water; (2) extraction under pressure; (3) extraction assisted by an enzyme/pectinase. The obtained extracts were analyzed in the UV-Vis domain, which allowed their characterization by determining the total monomeric anthocyanins, color density (control), polymeric density and browning index. The HPLC-MS analysis highlighted the extracts composition. The colors characteristics were determined through CIELab measurements. The performances of these 3 extracts, during green dyeing (without mordants), were evaluated according to the color characteristics (L*, a*, b* and K/S) of the dyed wool samples under different conditions: pH, temperature, duration of dyeing and volume of extract and stabilizers (Vitamin E and EDTA). Betalains can be considered acid dyes, with a low affinity for wool, which in a pronounced acidic environment dye the wool in an intense, uniform way and with good resistance to washing and rubbing.

## 1. Introduction

The last decade has seen a strong comeback of natural dyes as an alternative to synthetic dyes that can cause health and pollution problems. Synthetic dyes are very important compounds for the textile industry, but many of them are toxic, pollute wastewater, and cause allergies or other health problems to human factors [1]. The toxicity of synthetic dyes, confirmed by their presence in the REACH list (Registration, Evaluation, Authorization and Restriction of Chemicals Regulation) [2], has led to their exclusion more and more from use and to the general need to find ecological substitutes, such as natural dyes [3,4]. The alternative is therefore to resort to nature, as it offers a huge diversity of plants with colored parts (fruits, vegetables, flowers, rhizomes, tree bark, leaves, rinds, hulls, husks) that can be used as sources for the extraction of natural dyes covering the entire range of colors, starting with yellow, blue, red and any possible combinations. For example, sources like madder, brazilwood, beetroot, Morinda, black carrots or purple potatoes are feasible alternatives to synthetic red dyes [1,3,4]. What differentiates the pigments that provide the red colors from these sources are their chemical structures, so the classes to which they belong: betalains in the case of red beets and respectively anthocyanins in all the other listed sources [5,6]. Each natural pigment is valuable having a variety of biological properties and respectively a chromophore that ensures its color. These characteristics widen the scope of use of natural pigments: from the food industry and medicine to the textile industry. Retrieving information on the behavior of pigments during the processing of food or drugs (pH, time, temperature) is particularly useful, but they are not sufficient for dyeing textile materials because most natural pigments have no affinity for textile fibers; dyeing becomes possible if a mordant is used or when pigments or even textiles have been previously functionalized [7].

*Beta vulgaris* L. (beet) is a well-adapted, biennial plant belonging to *Amaranthaceae* family, that is easily accessible all over the world (Asia Minor, the Mediterranean, and Europe) [8,9]. It can be used as source for many biologically active substances, including betalains (betacyanins and betaxanthins), a group of highly bioactive pigments, as well as flavonoids (rutin, astragalin, kaempferol), terpenoids, saponins, phenolic acids, steroids, alkaloids, vitamins, tannins, sugars. Red-violet dyes of the betacyanin (betanin, E 162) type can be extracted from red beetroot (*Beta vulgaris* L.) [7,8]. The sub-species *Beta vulgaris* L. ssp. *vulgaris* contains 4 types of beets: fodder beet, sugar beet, garden beet/red beet and leaf beet (Swiss chard); of these only the last 2 can be used as vegetables and significant betalains and phenolic acids sources. Fodder beet is used as food for animals, while sugar beet is a source of sucrose, bioethanol, biodegradable polymers and biostimulants [8,9,10,11].

In Romania, the garden beet/red beet is an intensively cultivated vegetable, which is why its use for obtaining red-violet dyes has a very high potential. Although betalains is preferentially used in the food industry and medicine, recent research has shown that beetroot extracts can be used in the textile industry to ecologically dye all protein materials (with Oeko-Tex label) [7,12].

Betalains can be extracted either from the red beet pulp or from its peels, the latter being 4 to 7 times richer in betanin (the main red color component) depending on the maturity degree of the vegetable, the variety and the climatic conditions [7,13,14,15]. Along with betanin, other components of beetroot peel are: isobetanin, prebetanin, neobetanin, vulgaxanthin I, vulgaxanthin II, indicaxanthin, cyclodopa glucoside, glucoside of hydroxyindole-carboxylic acid, betalamic acid, L-tryptophan, p-coumaric acid, ferulic acid and traces of unidentified flavonoids were detected [15,16,17]. Betanin has no affinity for textile materials, so it does not determine their coloring. Furthermore, together with the other components of betalains, possess a high sensitivity to temperature [18,19,20] and pH [7,21,22], reason why the stability of betalains must be taken into account both in the extraction and dyeing processes [7]. The stability of these dyes depends on numerous dye-specific and external factors, as follows: the content of extract, high degree of glycosylation, high degree of acylation, pH, antioxidants, chelating agents, temperature, darkness, nitrogen atmosphere [7,14,15]. Modifying these factors, the color of the dyes is often affected, positively or negatively. In this context, the need to change betalains stability in order to generate new colored compounds that have an affinity for textile materials emerges.

In our previous work, we demonstrated that wool can be dyed with extracts from the dissolution of betalains from red beet peels in water, only if the betanin is subjected to functionalization in an acidic environment or if the wool is functionalized with acetic acid, ethanol or arginine [7].

The purpose of this study is to indicate 3 ways of valorizing beet peels in various ecological betalains extraction processes, identifying the components of the extracts and quantifying their performances in wool dyeing. The extracts come from sustainable pro-cesses based on: (1) dissolving betalains in water (REx); (2) extraction under pressure (PresEx); (3) extraction assisted by an enzyme/pectinase (PecEx). The characterization of the extracts and the identification of their basic components were carried out by performing UV-Vis, HPLC-MS and CIELab measurements.

The mechanisms underlying these sustainable extraction processes are, as follows:

1. water solubilization of any compound from betalains that has a good dissolving capacity (prebetanin, betanin/isobetanin, neobetanin, vulgaxanthin I/vulgaxanthin II, indicaxanthin [7]).
2. degradation of red beet peels of existing architectural construction (under the impact of high pressure and temperature) leads to create cracks that allow water to penetrate and solubilize easily soluble components.
3. pectinase acts only on pectin from red beet peels, which it hydrolyzes into polyga-lacturonic acid (pectic acid). Pectin is present in large proportions in red beet peels where it binds the different components ensuring the cohesion of the betalains; it turns out that during the extraction assisted by pectinase, the biomass of the red beet peels is degraded, which makes possible the penetration of water and the dissolution of easily soluble components.

The performances of these 3 extracts, during green dyeing (without mordants), are evaluated according to the color characteristics (L*, a*, b* and K/S) of the dyed wool samples under the conditions of varying pH, temperature, duration of dyeing, volume extract and respectively stabilizers (Vitamin E and EDTA). Fastness properties related to resistance to washing and rubbing were good, which indicates a high quality of dyeing wool with betalains.

## 2. Results and Discussion

### 2.1. UV-Vis

The UV-Vis analysis allowed the determination of the extract’s absorbance, at certain wavelengths, which made it possible to determine the characteristics of the extract’s colors. Table 1 shows the characteristics of REx1, PresEx1 and PecEx1 extracts calculated according to the relationships indicated by the literature [7].

**Figure 1:**
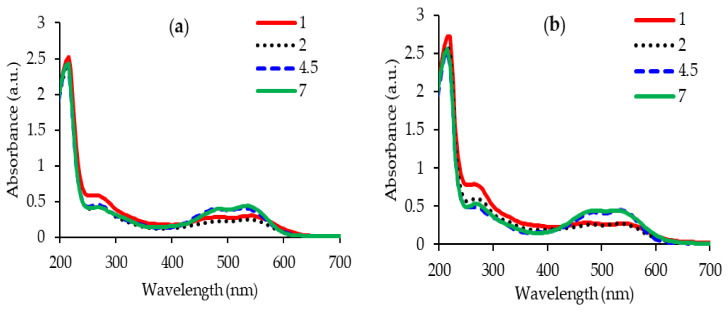
The influence of pH on the absorption of extracts: REx1 (**a**); PecEx1 (**b**).

The stability of betalains is dependent on several factors (pH, temperature, pres-ence/absence of oxygen, water and light) that can limit the use of extracts in the textile industry [23]. For this reason, in this study we focused on the pH dependence of the extracts, which influences both λmax and the maximum absorption intensity of betacyanins (Figure 1). The pH values of the extracts will also influence the colors of the wool samples dyed with these extracts.

The extracts stored for 1 h (REx1 and PecEx1) are characterized by hypsochromic and hypochromic effects. The hypsochromic effect consists of a blue shift, i.e., the absorption maximum is shifted to shorter wavelengths, by 5–15 nm, as the pH values increase:

1. for REx1: 540 nm at pH = 1; 535 nm at pH = 2; 530 nm at pH = 4.5; and 530 nm at pH = 7;
2. for PecEx1: 540 nm at pH = 1; 535 nm at pH = 2; 535 nm at pH = 4.5; and 525 nm at pH = 7;
3. for PresEx1: 540 nm at pH = 1; 535 nm at pH = 2; 535 nm at pH = 4.5; and 525 nm at pH = 7.

The hypochromic effect consists in reducing the maximum absorption intensity as the pH decreases from 7 to 1. Most likely this effect occurs due to the distortion of the geometry of the betalains molecules with an introduction/disappearance of new groups [7]. The degradation of betanin in a strongly acidic environment (pH = 1) involves the breaking of the ether bond between glycosides and betanidin with the release of the 2 components: glucose and betanidin [24,25,26,27].

In this work, the results of the hydrolysis processes generated by the acid environment on the extracts are observed on the UV-Vis spectra at 540 nm for the aglycone forms (betanidines and their deprotonated forms), 270 nm for cyclo-dopa-5-O-glycosides and 424 nm for betalamic acid [7,24]. The degradation of betalains with the release of cyclo-dopa-5-O-glycoside is confirmed by the increase in absorption intensity at 270 nm, in an acidic environment, compared to that at pH = 7. At pH = 2, the main components of betalains that ensure the red and yellow shades appear on the UV-Vis spectra at λmax = 535 nm for betanin and respectively at 480 nm for indicaxanthin. Additionally, a REx extract acidified to pH = 2 has betanidin, confirmed by HPLC-MS analysis, at retention time RT = 4.74. Another phenomenon that occurs in an acidic environment is the deprotonation of the carboxylic groups attached to C17, C15 and C2 or the deprotonation of the OH group attached to C6. At pH = 1, only the COOH group linked to C17 is deprotonated, and as the pH value increases, the number of COOH that releases protons increases, reaching 2–3 deprotonated carboxylic groups [7,27,28].

Compared to REx1, PecEx1 extract is richer in both betacyanins and betaxanthin; red-violet dyes have maximum absorption between 530–540 nm and yellow-orange dyes (indicaxanthin) absorb at approximately 480 nm, regardless of the pH value used in the extraction process (Figure 1). However, the pH influences the intensity of the maximum absorption: an acidic environment with pH = 2 generates a 1.7 times reduction compared to REx1 (with pH = 7) and 1.6 times compared to the case of PecEx1 (at pH = 7).

### 2.2. HPLC-MS

The HPLC-MS analysis shows the presence of betanin/isobetanin as main compound. Combining mass spectrometric with DAD detection provide total ion current (TIC) profiles of compounds eluting from the HPLC column, for peaks that have a particular color (disregarding extraneous, non-dye components, abundant in extracts of plant material), sometimes giving fragmentation patterns that yield even more structural information) (Table 2). The main component, betanin/isobetanin (551 on the MS spectrum) was identified in both extracts REx1 and in PresEx1 but REx1 extract also contains yellow dyes as Vulgaxanthin IV/Isovulgaxanthin IV (Figures S1 and S2 in the Supplementary Material). HPLC-MS analysis shows that the extract performed at a high temperature and pressure, PresEx1 contains besides betanin/isobetanin and decarboxylated forms of betanin/neobetanin, at the C17/C15/C2 level (Table 2). Taking into account information from the literature [29], which indicates that all decarboxylated forms provide lighter red colors with yellow shades, it follows that the PresEx1 extract will dye the wool in lighter colors.

### 2.3. Characterization of Betalains from Extracts

The main characteristics of the betalains identified in the REx and PresEx extracts are presented in Table 3.

### 2.4. Color Characterization of Extracts

**Figure 2:**
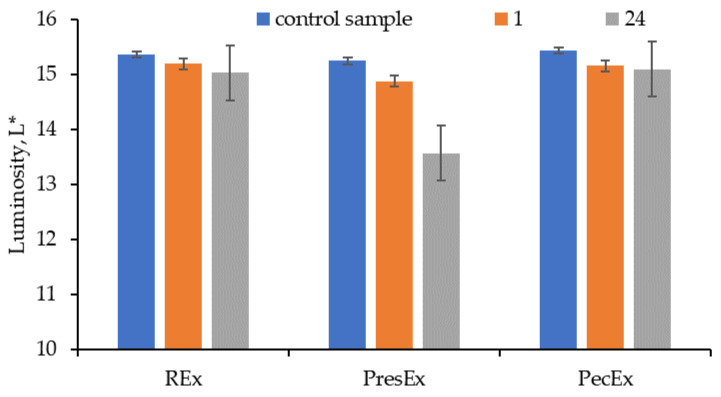
The luminosity of the extracts depending on the duration of the extraction process; (Conditions for obtaining: 100 mL protonated extract with acetic acid, 30 min, pH = 3.5). The figure was obtained at a level of significance α = 0.05.

**Figure 3:**
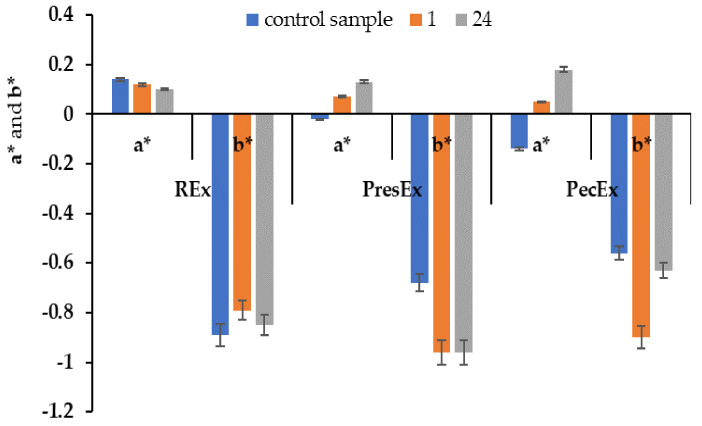
The a* and b* values for REx, PresEx and PecEx extracts, obtained from extractive processes with different durations (1–24 h). The figure was obtained at a level of significance α = 0.05.

The colors of REx, PresEx and PecEx extracts depend on the duration of the extraction process: extending the extraction process up to 24 h leads to the intensification of the red color, especially in the case of PresEx24 (Figure 2 and Figure 3).

The red color of each extract changes as the storage time increases. In the case of REx, after 24 h the value of a* decreases from 0.14 to 0.1; after 24 h the bluish shade of the red extract diminishes, a fact confirmed by the change in the value of b* from −0.89 to −0.85. In the case of PresEx, a* increases from −0.02 to 0.13, but the yellow shade from the red color of the extract decreases and the blue tint intensifies (initially, b* being −0.68 and after 24 h of storage it becomes −0.96). In the case of PecEx, a* increases from −0.14 to 0.18, but the yellow shade from the red color of the extract decreases and the blue one intensifies, this phenomenon being less intense than in PresEx because the control sample/extract has b* = −0.56 and after 24 h it has b* = −0.63.

### 2.5. Wool Dyeing Assisted by Sustainable Extracts

The mechanism of wool dyeing with REx24, PresEx24 and PecEx24 extracts, in an acidic medium, is based on the stages of deprotonation, wool activation and dyeing [7]. The deprotonation of betalains consists in the formation of COO-type anionic groups, a phenomenon intensified as the pH approaches the value 7. The second stage, the activation of the wool occurs due to the acidic environment in the dyeing bath; ^+^NH_3_ type cations are formed in the wool, which interact with the anions in the acid that ensure the pH of the dye bath, forming ionic bonds. The third stage, the actual dyeing takes place at temperature (between 30 and 70 °C), when the betalains molecules replace the acid anion from the ionic bonds formed in the activated wool.

The factors that influence the color of wool samples dyed with extracts resulting from long extraction processes (24 h), were pH, stabilizers (Vitamin E and EDTA), dyeing duration (24, 25 and 26 h at room temperature or 1 h at 40, 50 or 70 °C), dye concentration (25–100 mL extract), and dyeing temperature (40, 50 and 70 °C).

#### 2.5.1. Influence of pH on Dyeing Wool

To determine the influence of pH on dyeing wool with red beetroot peel extract, both the stability of the extract in the dye liquor and the effect of the acid on the wool must be taken into account. Another factor would be the involvement of temperature/pressure in the extraction process (as with PresEx).

The stability of the betalains in the extract depends on the pH—temperature correlation, a fact confirmed by the literature which indicates that at low temperature, betalains from the beetroot juice are stable in an acidic environment, but the optimum pH is 4–6 [32]. The same pH-temperature correlation also influences the stability of the extracts used in wool dyeing, which can be seen from the shades obtained on the dyed samples. During the heating of the acidified extracts (i.e., of the dyeing fleet), decarboxylation, dehydrogenation and deprotonation phenomena occur [7,23,33,34]. The phenomenon of decarboxylation of betanin with the formation of 15-decarboxy-betanin, 17-decarboxy-betanin/isobetanin is present in any acidic environment, but the 2-decarboxy-betanin compounds are largely formed only when the acidic environment is more pronounced (pH 3–4) [23,33]. The decisive factors that determine the formation of 2-decarboxy-betanin/isobetanin are the concentration of betanin/isobetanin, the nature and concentration of the acid used to create the pH: a low concentration of acetic acid (1 g/L) is preferable, while the addition of ethanol determines the obtaining of 17-decarboxy-betanin/isobetanin compounds [34]. Other betanin derivatives (such as 2-decarboxy-xanbetanin and 2-decarboxy-neobetanin) are formed due to heating and/or an acidic environment (pH = 3–5, as in the PresEx24 extract) by dehydrogenation or by dehydrogenation + decarboxylation reactions [23,33]. The compound 2-decarboxy-xanbetanin results from the dehydrogenation + decarboxylation of betanin, while the compound 2-decarboxy-neobetanin results from the decarboxylation of neobetanin; both compounds can result from dehydrogenation of 2-decarboxy-betanin, under certain conditions.

**Figure 4:**
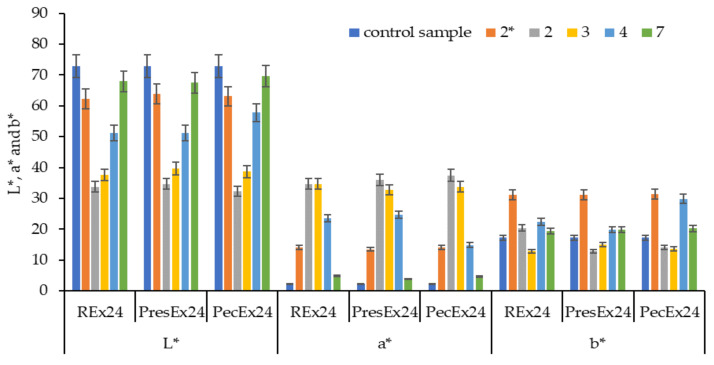
Influence of pH on CIElab values of wool samples dyed at 30 °C, for 24 h (Legend: 2* represents a pH = 2 made with H_2_SO_4_, the other acidic environments being made with acetic acid). The figure was obtained at a level of significance α = 0.05.

**Figure 5:**
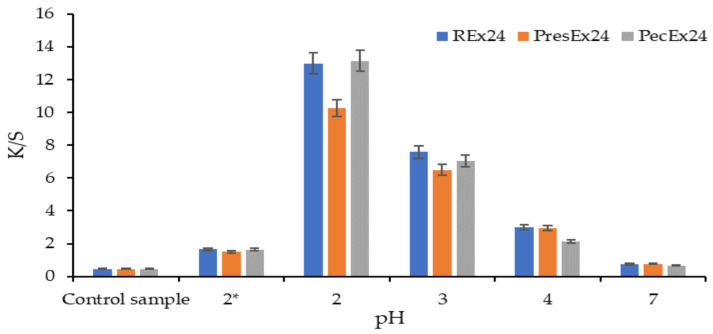
Influence of pH on color intensity (K/S) values of wool samples dyed at 30 °C, for 24 h; (Legend: 2* represents a pH = 2 made with H_2_SO_4_, the other acidic environments being made with acetic acid). The figure was obtained at a level of significance α = 0.05.

**Figure 6:**
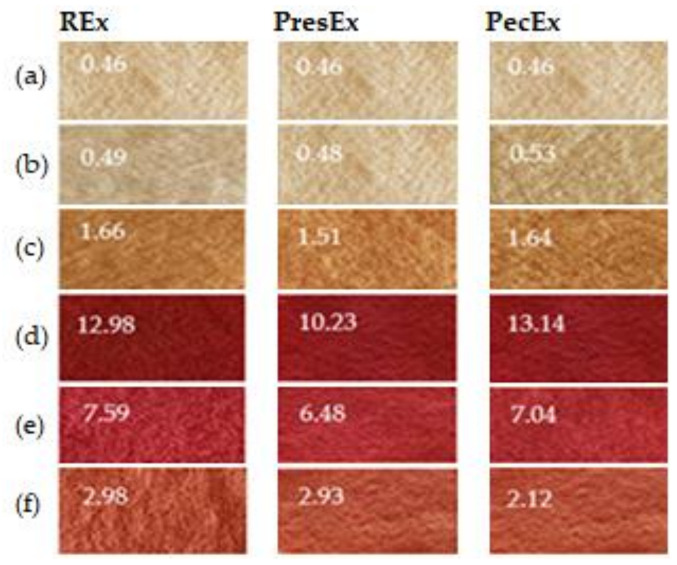
Appearances and the color intensities for wool samples before dyeing (**a**), dyed for 4 h with colored extracts, without acid addition (**b**); dyed 4 h with colored extracts acidified with H_2_SO_4_ up to pH = 2 (**c**); dyed with REx24/PresEx24/PecEx24 at pH 2 (**d**), pH 3 (**e**), pH 4 (**f**); (Dyeing conditions: 100 mL extract, for 4–24 h at 30 °C (room temperature, summer)).

Regardless of the temperature value, the acidity of the dyeing medium influences the deprotonation of betalains and the activation/protonation of wool, without which dyeing cannot take place. These interdependencies led us to test a larger range of pHs (2–7), at a temperature of 30 °C of the dyeing fleet. Strongly acidic dye baths (pH = 2) made with acetic acid (pKa = 4.75) in the colored extracts (REx24/PresEx24/PecEx24) lead to the lowest luminosities (L* = 32.03–33.76) and to highest values for a* (a* = 34.79–37.47). In these cases, the wool is dyed intensely red because at this pH, the deprotonation of a carboxylic group from betanin takes place, which makes it possible to create an electrovalent bond with the cationic group in the wool activated by the acid environment in the dyeing bath. The increase in the pH value between 2 and 7 determines the increase in the luminosity of the extracts and respectively the decrease in the red shades of the dyed samples. In our previous work [7] we showed that as the pH increases, the number of COO^−^ groups in betalains also increases, as a result of the deprotonation phenomenon, which occurs at carbon atoms C17, C15 and/or C2. However, when a large number of deprotonated COO^−^ type groups from betalains (at pH values close to 7) are simultaneously attracted to the same wool dyeing center (^+^NH_3_ group) then it becomes difficult to form an electrovalent bond, due to the competition of the COO^−^ groups, which determines an insufficient proximity of the groups of opposite signs that would allow the formation of the ionic bond. Using a strong inorganic acid (H_2_SO_4_) to achieve pH = 2 leads to yellow-orange colors. The sulfuric acid (pKa = −2.8 and 1.99) being a stronger acid than betanin (pka =1.46) will block betanin deprotonation. H_2_SO_4_ will give up a proton that will contribute to the protonation of NH_2_ groups from vulgaxanthin I; this cationic group has affinity for the COO^−^ groups from the wool, so it will color it yellow-orange because the specific color of this bethaxanthin/vulgaxanthin I is yellow-orange (Figure 4, Figure 5 and Figure 6).

Figure 4, Figure 5 and Figure 6 indicate that an inorganic acid such as sulfuric acid has a negative influence on the coloring of the wool with the colored extracts studied. Instead, the highest color intensities are obtained at pH = 2 obtained with acetic acid, followed by those at pH = 3 and then at pH = 4. In the presence of acetic acid, the REx24 extract can be considered the most suitable for dyeing wool, regardless of the pH of the dyeing bath because this extract is the easiest to apply, it is more economical to obtain because it is based only on the dissolution of betalains in water, without the presence of enzymes or other solvents that can be expensive. In addition, the color intensities of wool dyed with REx24 are higher than in the case of PecEx24. The extract obtained at high temperature and pressure, the PresEx24 extract leads to the lowest color intensities because during the extraction process, the high temperatures cause the decarboxylation phenomenon of betalains to occur (phenomenon confirmed by the results of the HPLC-MS analysis). The loss of some COOH groups from C17, C15 or C2 in betalains leads to much weaker red colors than non-decarboxylated betalains can give. In the case of PresEx24, the mono-deprotonated and 2-decarboxy-betanin/isobetanin forms (responsible for the coloring) are formed in large quantities when the pH is 2–4. The forms 2-decarboxy-neobetanin appear at pH = 3–4 from the conversion of neobetanin and 2-decarboxy-xanbetanin appears from the conversion of betanin at pH = 5–6 [34].

#### 2.5.2. Wool Dyeing in the Presence of Stabilizers

The literature indicates studies in which betalains stability was tested in the presence of ascorbic acid [35], isoascorbic acid [18,36,37], citric acid and EDTA [18,38]. Stabilizers such as Vitamin E and EDTA were used to prevent the phenomenon of oxidation, browning or diminution of the color of the extracts. These compounds stabilize the color of the extracts by acting through antioxidation and/or chelation mechanisms, offering the possibility of producing dark red colors on woolen materials during dyeing.

The stability of betanin in extracts is higher than in pure solutions because the extracts also contain polyphenols that have an antioxidant effect. However, the presence of metals accumulated by beets during growth can cause the catalysis of the oxidation reactions of some components of betalains (betanin, 2-decarboxy betanin, 17-decarboxy-betanin and 2,17 bidecarboxy-betanin) with the formation of neo-derivatives respective xan-derivatives of betanin [34]. The effect of these oxidations consists in the change in betanin color observable through blueshift (hypsochromic effect) and the reduction of absorption intensity from λmax (hypochromic effect) [34,39,40]. For these reasons, it is not recommended to use metals or metal salts as mordants in the dyeing of textile materials with betalains.

Another explanation for the loss of color of the extract in the presence of metals is based on the formation of complexes of the metal-betalains type. To prevent the formation of these complexes and to avoid unwanted color degradation phenomena, there are 2 possibilities: adding a chelating agent and acidification [39,40,41]. Adding a chelating agent such as EDTA (pKa = 0.26, which can easily deprotonate) will chelate all the metal ions in the extract. This eliminates the risks of interaction of metals with betanin (through the newly deprotonated COO^−^ groups from carbon atoms 17 and C-15, at low pHs) and the formation of metal-betanin complexes. Acidification to a low pH causes the destruction of the metal-betanin complexes because the hydrogen ions, in excess, enter into competition with the metal ones, which reduces or even eliminates the unwanted complexation phenomenon [40]. In the case of EDTA, the best stability effects on betanin were recorded at pH = 2 and at pH = 5 [39].

**Figure 7:**
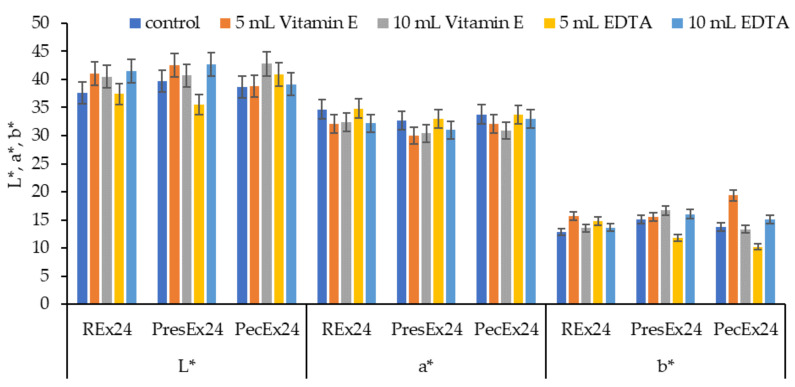
The influence of stabilizers on the color of wool dyed 24 h at 30 °C, pH = 3.5 with 100 mL extract. The figure was obtained at a level of significance α = 0.05.

In the acidic environment of the dyeing bath, the COO^−^ groups in the dye will form electrovalent bonds with the ^+^NH_3_ groups in the activated wool, a fact confirmed by dyeing the wool in intense colors. Dyeing wool with 100 mL extract, at pH = 3.5 (made with acetic acid), for 24 h at room temperature (30 °C, room temperature in summer), in the presence of 5 mL EDTA led to red colors intense characterized by a* = 34.83 at Rex24, by 33.01 at PresEx24 and 33.73 in the case of PecEx24 (Figure 7).

**Figure 8:**
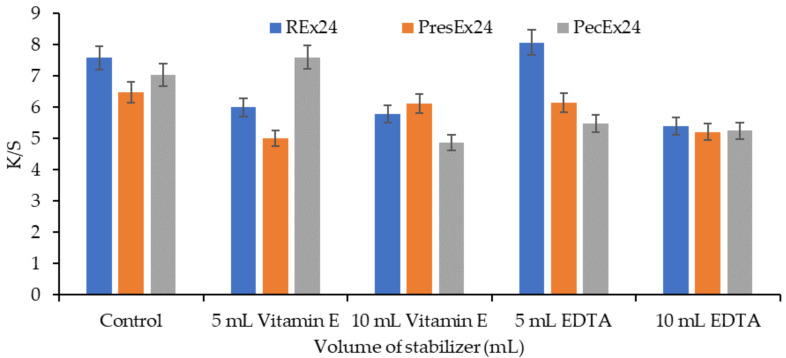
The influence of stabilizers (Vitamin E and EDTA) on color intensity when dyeing wool with 100 mL extract + 5–10 mL stabilizer, at pH = 3.5, T = 30 °C for 24 h. The figure was obtained at a level of significance α = 0.05.

**Figure 9:**
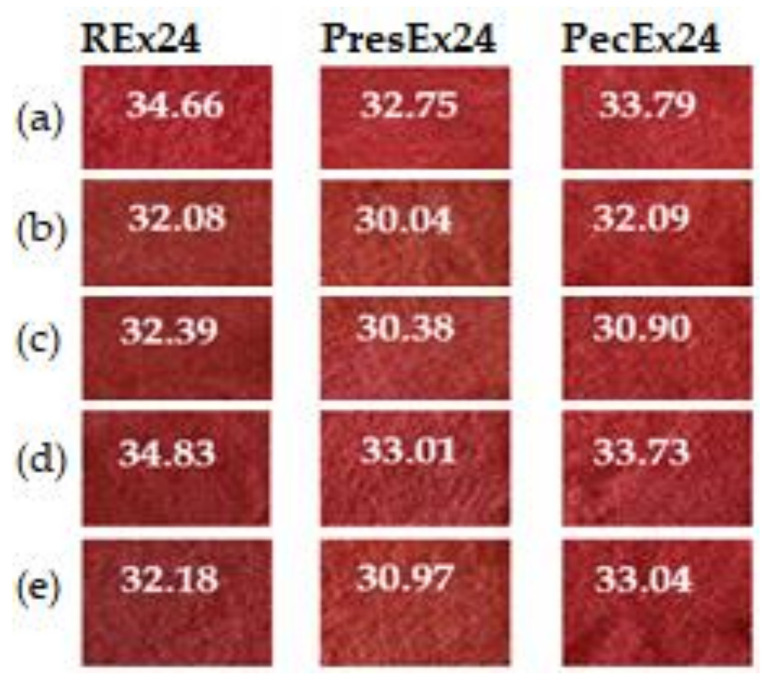
Appearances and a* values of wool samples dyed with 100 mL extract at pH = 3.5, T = 30 °C for 24 h, without stabilizer (**a**) and with stabilizer: 5 mL Vitamin E (**b**), 10 mL Vitamin E (**c**), 5 mL EDTA (**d**) and 10 mL EDTA (**e**).

Figure 7 shows that the best stabilization effects were obtained with 5 mL EDTA in Rex24 and PresEx24 extracts, when compared to the standard sample, L* values decreased and a* values increased. In REx24, L* decreased from 37.6 to 37.41 and the a* value increased from 34.66 to 34.83. In PresEx24, L* decreased from 39.67 to 35.54 and the a* value increased from 32.75 to 33.01. In PecEx24, EDTA has no stabilizing effect, but a small volume of Vitamin E (5 mL) results in keeping L* and a* close to the values of the control sample. However, the stabilizing effect of Vitamin E on the color is highlighted by the increase in K/S values, from 7.044 to 7.605, because the red color is the combination of the red given by betacyanins (a* = 32.09) and the yellow given by of betaxanthins (with high b* values of 19.39) (Figure 8 and Figure 9).

#### 2.5.3. Influence of Extract Volume

**Figure 10:**
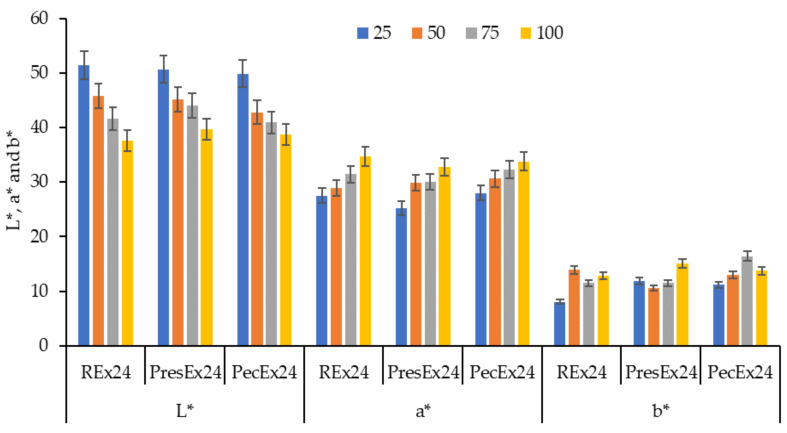
The influence of the extract volume (25–100 mL Rex24, PresEx24 or PecEx24) on the color characteristics (L*, a* and b*) of wool dyed 24 h at pH = 3.5 and T = 30 °C. The figure was obtained at a level of significance α = 0.05.

Increasing the volume of the extract in the dyeing bath (from 25 to 100 mL extract) determines a more intense coloring of the wool, a fact confirmed by the changes in the CIELab sizes: the decrease of L* values, the increase of a* (Figure 10).

**Figure 11:**
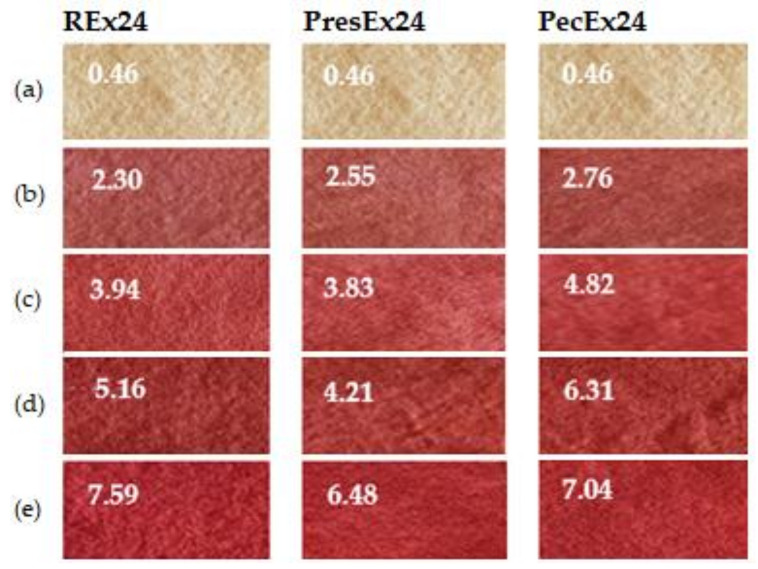
Appearances and the color intensities of wool samples before dyeing (**a**), after dyeing with a certain volume of extract: 25 mL (**b**), 50 mL (**c**), 75 mL (**d**) and 100 mL (**e**); (dyeing conditions: 24 h at pH = 3.5 and T = 30 °C).

**Figure 12:**
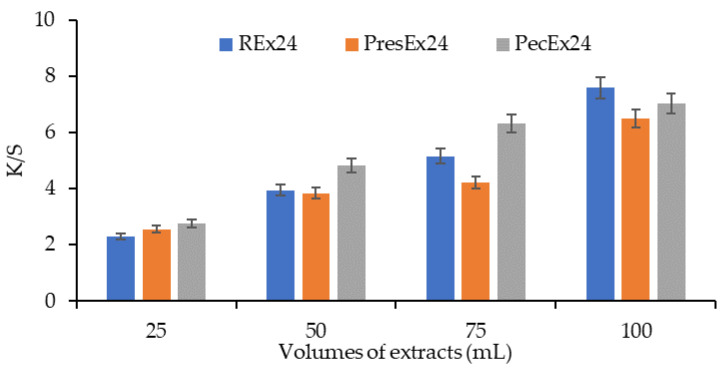
Dependence of the color intensity of wool dyed with 25–100 mL Rex24, PresEx24, and PecEx24 extracts, at T = 30 °C and pH = 3.5. The figure was obtained at a level of significance α = 0.05.

The K/S values increase as the volume of extract placed in the dyeing bath is larger (Figure 11 and Figure 12). The dyeing mechanism is based on the formation of electrovalent bonds between the wool dyeing centers (that is, the ^+^NH_3_ groups in the wool activated by the acetic acid added to the dyeing bath to achieve pH = 3.5) and the deprotonated carboxylic groups in betanin.

#### 2.5.4. The Temperature Influence

**Figure 13:**
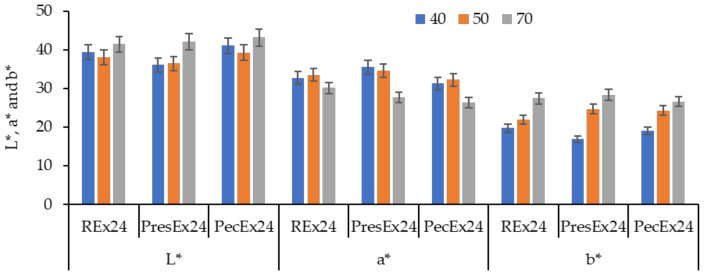
The influence of the dyeing temperature (40–70 °C) on the color characteristics of wool dyed with 100 mL extract, at pH = 3.5, t = 1 h. The figure was obtained at a level of significance α = 0.05.

In general, betanin stability decreases with increasing temperature, but it also depends on the prevailing conditions (pH, duration, concentration of betalains) usually leading to mono-decarboxylated (at C2, C15 and C17) and bi-decarboxylated forms (at C2 and C17 or at C2 and C15) [33,42,43] or even to neobetanin and betalamic acid compounds, in the case of advanced degradations [18,19,33]. Temperature is the factor that determines the decrease in the extracts color (respectively the increase of the luminosity L*, the decrease of the red hue by decreasing the values of a* and the increase of the values of b*) due to the degradation of betalains by dehydrogenation and decarboxylation (Figure 13). The occurrence of the decarboxylation phenomenon at the level of carbon atoms C17, C12 and C2 was confirmed by the results of the HPLC-MS analysis in the case of the extract obtained at high pressure and temperature, PresEx (Table 1). The mechanism consists in the autoxidation of betanin (initiated by heating) which leads to 15-decarboxy-betanin, 17-decarboxy-betanin/isobetanin and 2-decarboxy-betanin; the last compound can be dehydrogenated and generate 2-decarboxy-xanbetanin, which can also be obtained by transforming quinone methide following a dehydrogenation and decarboxylation reaction at the C2 carbon atom [44,45]. In general, the compounds 15-decarboxy-betanin, 17-decarboxy-betanin/isobetanin are constantly obtained from the conversion of betanin in the entire pH range tested, but the compound 2-decarboxy-betanin is largely formed only when the environment is more acidic (pH 3–4) [33].

**Figure 14:**
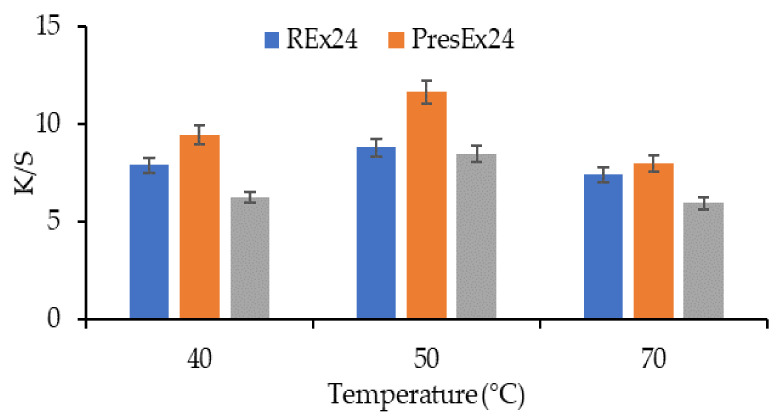
Dependence of the color intensity of wool dyed at 40–70 °C, with 100 mL extracts, at pH = 3.5 (CH_3_COOH), t = 1 h. The figure was obtained at a level of significance α = 0.05.

**Figure 15:**
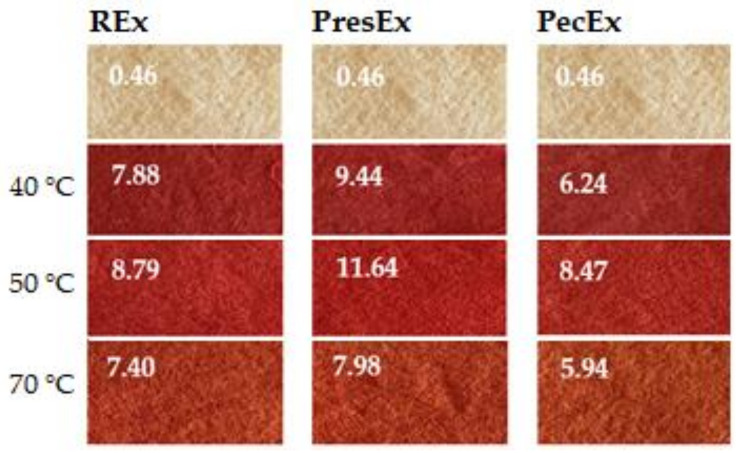
Appearances and the color intensities of dyed wool samples at 40–70 °C, with 100 mL extracts, at pH = 3.5 (CH_3_COOH), t = 1 h.

Figure 14 and Figure 15 indicate that 50 °C can be considered the optimal dyeing temperature, because it leads to higher K/S values than at 40 °C and 70 °C, regardless of the type of extract used as dyeing stock.

#### 2.5.5. The Influence of Dyeing Duration

**Figure 16:**
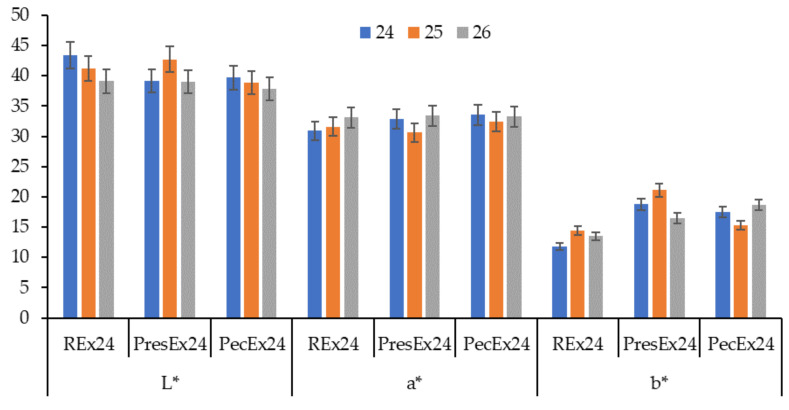
Dependence of L*, a* and b* on the duration of dyeing (24–26 h) if the dyeing is done at pH = 3.5, T = 30 °C with volumes of 100 mL extracts. The figure was obtained at a level of significance α = 0.05.

**Figure 17:**
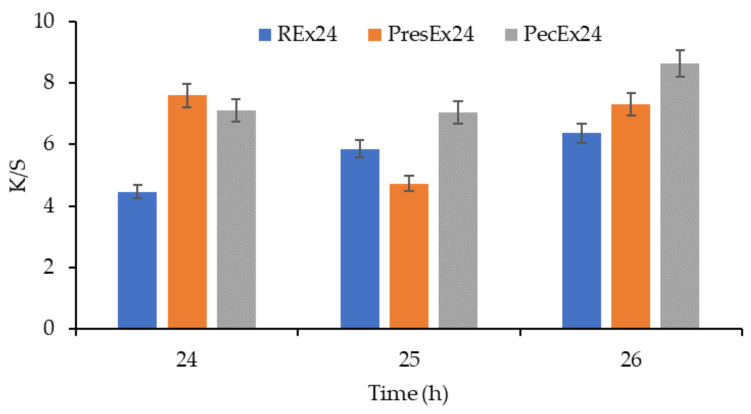
Dependence of K/S on the duration of dyeing with 100 mL extract, at 30 °C, pH = 3.5. The figure was obtained at a level of significance α = 0.05.

Dyeing the wool with fresh extract obtained from the peels of red beet does not lead to the coloring of the wool [7]. It is necessary to store/stand the extract for at least 24 h; during this time, phenomena occur that convert betalains into certain forms that have an affinity for wool. The decrease of L*, the increase of a* and K/S values indicate that the extension of the dyeing time leads to more intense coloring of the wool in the cases of using the REx24 and PecEx24 extracts (Figure 16 and Figure 17). In the case of PresEx24, extending the dyeing time by 1 h, from 25 to 26 h, results in an intensification of the coloring, a fact confirmed by the change in the luminosity values, L* from 42.72 to 39, the a* values from 30.67 to 33.4 and the intensity color K/S from 4.73 to 7.295.

#### 2.5.6. Fastness Properties

The quality of the dyed wool is reflected by the strength of the ionic interactions between the wool and the betalains. The 3 types of extracts used for dyeing the wool led to good fastness properties highlighted by the high values of the notes awarded for color fastness to washing and color fastness to rubbing (Table 4).

## 3. Materials and Methods

### 3.1. Plant Materials

The vegetables from the species *Beta vulgaris* L. were cultivated in Romania in the area of Iasi. Before use, the red beetroots were washed very well, then they were left to dry naturally for 24 h. The next steps were peeling the red beets and weighing them accurately (200 g) before entering them into the dye extraction processes.

### 3.2. Chemicals and Protein Material

Acetic acid 80%, sulfuric acid, ethylenediaminetetraacetic acid (EDTA) and DL-α-tocopherol acetate (Vitamin E) were purchased from Merck. The pectinase type enzyme was purchased from Novo Nordisk Bioindustries. The protein material used was wool (in the form of fabric with 250 g/m^2^).

### 3.3. Experimental Protocol

For the extraction of betalains, batches of 200 g of red beet peels in 2 L of distilled water were used, proceeding in 3 ways:

1. without other additions to assist the extraction; the phases were maintained for 1 or 24 h at room temperature to complete the betalains extraction. The raw extracts obtained (abbreviated REx1 or REx24) were used to dye wool samples in 100 mL extract, at different pHs (2–7), temperatures (30–70 °C), durations (1–26 h), or in the presence of 5–10 mL stabilizer (Vitamin E respectively EDTA). The control sample was considered the extract obtained after 10 min of solubilizing and shaking the red beet peels in distilled water.
2. extraction under pressure (at 105 °C, for 10 min) and keeping the extract for 1 h or 24 h to complete the reaction. The obtained extracts were named “PresEx1 and PresEx24 respectively”. Control sample was considered the PresEx extract after 10 min from the completion of the extraction process.
3. enzyme-assisted extraction (2 mL commercial pectinase in 2 L water), at room temperature, for 1–26 h; the obtained extracts were abbreviated PecEx1 and PecEx24 respectively. The control sample was considered the extract stored 10 min after the completion of the extraction process.

All three types of extracts obtained after 24 h were used to dye wool samples of 0.5 g using 25–100 mL extract each at 30–70 °C, for 1–24-26 h, pHs (2–7) or in the presence of 5–10 mL stabilizer (Vitamin E or EDTA).

### 3.4. Analysis

#### 3.4.1. UV-Vis

The UV–Vis spectra were performed in the 200–800 nm range on a Single Beam Scanning M 501 UV–Vis spectrophotometer. This analysis allowed the characterization of extracts, at different wavelengths and at different pHs. In accordance with the relationships presented in our previous work [7], the following color characteristics of the extracts were determined: total monomeric anthocyanins, color density (control), polymeric density, browning index.

#### 3.4.2. HPLC-MS Experimental Technique

For the identification of the compounds in the beetroot extract a Thermo HPLC-MS system was used equipped with a Diode Array and MSQ Plus detector, an Acclaim 120 C18 column (150 mm × 4.6 µm), acetonitrile (5–95%) and formic acid 1% (95–5%), with a flow rate of 0.4 mL/min as mobile phase and 10 µL injection volume. The MSQ plus functioned on ESI mode (positive ion mode), with 250 °C sample temperature, and +75 V (cone). The UV absorptions were recorded at 4 wavelengths: 310 nm, 480 nm, 505 nm and 541 nm.

#### 3.4.3. Colorimetric Measurements

The color characterization of the extracts and of the dyed samples was carried out with the help of a Datacolor Sprectroflash SF300 spectrophotometer on which CIEL*a*b* measurements were performed. The meanings of the color characteristics are, as follows: L* is the luminosity; a* indicates the color position of the dyed samples, on the red-green axis and b* on the yellow-blue axis, on the CIEL*a*b* color space diagram. The Datacolor Sprectroflash SF300 spectrophotometer also indicated the K/S color intensity values.

#### 3.4.4. Error Bars to Excel Graphs

All extractions and dyeing processes were performed in triplicate and the graphs were plotted using the mean values of the obtained results. Microsoft Excel allowed percentage error bars to be determined and added to each graph when a percentage error range was specified (in this study 5% was used). Error bars in Excel are graphical representations of data variability. They show the precision of a measurement, indicating how far the true value is from the determined value. A short error bar indicates that the values are concentrated, signaling that the average value represented is more likely. A long error bar indicates that the values are more spread out and less reliable.

#### 3.4.5. Fastness Properties

Fastness properties were determined by performing the tests ISO 105-C10: 2010 (as Color fastness to washing test) and ISO 105-X12 (as Color fastness to rubbing test). For both tests, a gray scale was used for assessing staining of the undyed lining fabrics. In the case of washing test, a gray scale was also used to assess the discoloration of the dyed sample, due to washing.

## 4. Conclusions

The color of the betalains is often affected by the factors as acidic environment, stabilizers, volume of the extract, duration and temperature of dyeing. The acidic environment made with acetic acid determines the deprotonation of betanin, which gains affinity for the ^+^NH_3_ groups in the activated wool, considered to be the future dyeing centers; the lower the pH, the more intensely the wool is colored, because in a strongly acidic environment, only mono-deprotonated forms of betalains are formed that can get close enough to the dye sites in the wool so as to form ionic bonds. This is more difficult in weaker acid environments when the deprotonation phenomenon is favored resulting di-deprotonated and/or tri-deprotonated forms; these betalains, having several COO^−^ groups, are simultaneously attracted to different wool dyeing centers, not allowing them to get close enough to ensure the distance necessary for the formation of ionic bonds. It is advisable to use chelating agents because they can act as stabilizers, eliminating the risk of forming metal-betanin complexes or even regenerating the color of the extracts when the environment is strongly acidic. The best stabilization effects, with positive effects on the K/S values of the dyed wool, are observed in the presence of 5 mL of EDTA in REx24/PresEx24 and with the addition of 5 mL of Vitamin E in PecEx24. Regardless of the presence or absence of stabilizers, increasing the volume of the extract (from 25 to 100 mL) and the duration of dyeing (24–26 h) have positive effects on the color intensity of the dyed wool samples. Conversely, the increase in dyeing temperature causes the degradation of betanin to decarboxylate forms (red-yellow) or even to betalamic acid (yellow) which causes a decrease in the color intensity of the dyed wool. Among the decarboxylated forms occurring at carbon atoms C2, C15 and C17, only the compound 2-decarboxy-betanin has the greatest contribution in ensuring the red color of the wool, especially in the case of using the PresEx24 extract for dyeing. In this context, the results prove that the altering of betalains stability from the red beetroot leads to new colored compounds that may have affinity for textile materials.

## Figures and Tables

**Table 1:** Characterization of extracts (REx1, PresEx1 and PecEx1).

Extract *	Total Monomeric Anthocyanins (mg/L)	Color Density	Polymeric Color Density (Bisulfite)	Browning Index
REx1	142.35	0.5976	0.7695	0.2046
PresEx1	135.23	0.5600	0.7300	0.1900
PecEx1	142.71	0.6181	0.8057	0.0847

* The number 1 associated with each extract indicates the duration of the extraction process.

**Table 2:** Retention times of compounds determined by HPLC-MS analysis.

Sample	Compound	RT (min)	*m*/*z* [M + H]^+^
REx1	Betanin/Isobetanin	3.70	551
	Vulgaxanthin IV/Isovulgaxanthin IV	9.49	325
PresEx1	2-decarboxy-Neobetanin/2-decarboxy-Xanbetanin	2.77	505
	15-decarboxy-Betanin 17-decarboxy-Betanin/17-decarboxy-Isobetanin	3.18	507
	2-decarboxy-Betanin/2-decarboxy-Isobetanin	3.57	507
	17-decarboxy-Isobetanidin	3.88	345
	Betanin/Isobetanin	3.89	551

**Table 3:** Characterization of the main compounds present in extracts [30,31].

Property	Betanin	Isobetanin	2-decarboxy-betanin	2-decarboxy-neobetanin	15-decarboxy-betanin	17-decarboxy-betanin	17-decarboxy-isobetanin	Vulgaxanthin IV
Compound no. in [26] *	FDB000497	-	FDB015256	-	-	-	-	-
Compound no. in [27] **	-	PHUB000406	-	PHUB000412	PHUB000416	PHUB000413	PHUB000414	PHUB000435
Synonyms	E 162	isobetanidin 5-O-beta-glucoside	2-Descarboxy betanin	-	-	-	-	Leucine-betaxanthin
Chemical formula	C_24_H_26_N_2_O_13_	C_24_H_26_N_2_O_13_	C_23_H_27_N_2_O_11_	C_23_H_26_N_2_O_11_	C_23_H_27_N_2_O_11_	C_23_H_27_N_2_O_11_	C_23_H_27_N_2_O_11_	C_15_H_20_N_2_O_6_
Molecular weight (g)	550.4688	550.473	507.4673	506.464	507.471	507.471	507.471	324.333
Water solubility (g/L)	0.5	5.00 × 10^−1^	0.49	1.85	7.73 × 10^−1^	7.87 × 10^−1^	7.87 × 10^−1^	1.60 × 10^−1^
pKa (Strongest acidic)	1.46	2.402	3.22	3.008	2.669	0.9196	0.919	1.84
pka (Strongest basic)	−3.6	−3.648	−3	7.174	1.726	8.1168	8.116	8.81
Hydrogen acceptor count	14	14	12	13	12	12	12	8
Hydrogen donor count	8	8	8	7	8	8	8	4
Number of rings	4	4	4	4	4	4	4	1

* [30] https://foodb.ca/compounds/ (accessed on 4 March 2023). ** [31] https://phytohub.eu/entries/ (accessed on 4 March 2023).

**Table 4:** Results of fastness properties for wool samples dyed with REx24, PecEx24 and PresEx24 (Dyeing conditions: pH = 3.5 (CH_3_COOH), 100 mL extract, T = 30 °C).

Sample Code	Color Fastness to Washing			Color Fastness to Rubbing	
	Change in Color	Staining			
		Cotton	Wool	Dry	Wet
Rex24	5	5	5	5	4–5
PresEx24	4–5	5	5	5	4–5
PecEx24	5	5	5	5	4–5

## Data Availability

Not applicable.

## Supplementary Materials

The following supporting information can be downloaded at: https://www.mdpi.com/article/10.3390/plants12101933/s1, Figure S1: HPLC chromatogram (a) and mass spectra (b and c) of the compounds identified in the Rex1 extract; Figure S2: HPLC chromatogram (a) and mass spectra (b–e) of the compounds identified in the PresEx1 extract.
