# Supplementary material for: Beta vulgaris L.—A Source with a Great Potential in the Extraction of Natural Dyes Intended for the Sustainable Dyeing of Wool
Source: Plants (Basel). 2023 May 9;12(10):1933. doi: 10.3390/plants12101933 (PMC10222782; doi:10.3390/plants12101933)
Supplement: Supplementary file 1 [file plants-12-01933-s001.zip › plants-2354206-supplementary_9.05.2023.pdf]

Supplementary material
for

Beta vulgaris L.—A Source with a Great Potential in the Extraction of Natural Dyes Intended for the Sustainable Dyeing of Wool

Vasilica Popescu [1,*], Alexandra Cristina Blaga [2], Dan Cașcaval [2] and Andrei Popescu [3]

[1] Department of Chemical Engineering in Textiles and Leather, Faculty of Industrial Design and Business Management, “Gheorghe Asachi” Technical University of Iasi, 700050 Iasi, Romania
[2] Department of Organic, Biochemical and Food Engineering, “Cristofor Simionescu” Faculty of Chemical Engineering and Environmental Protection, “Gheorghe Asachi” Technical University of Iasi, 700050 Iasi, Romania; alexandra-cristina.blaga@academic.tuiasi.ro (A.C.B.); dan.cascaval@academic.tuiasi.ro (D.C.)
[3] Department of Mechanical Engineering, Mechatronics and Robotics, Faculty of Mechanical Engineering, “Gheorghe Asachi” Technical University of Iasi, 700050 Iasi, Romania; andrei.popescu@academic.tuiasi.ro
* Correspondence: vasilica.popescu@academic.tuiasi.ro

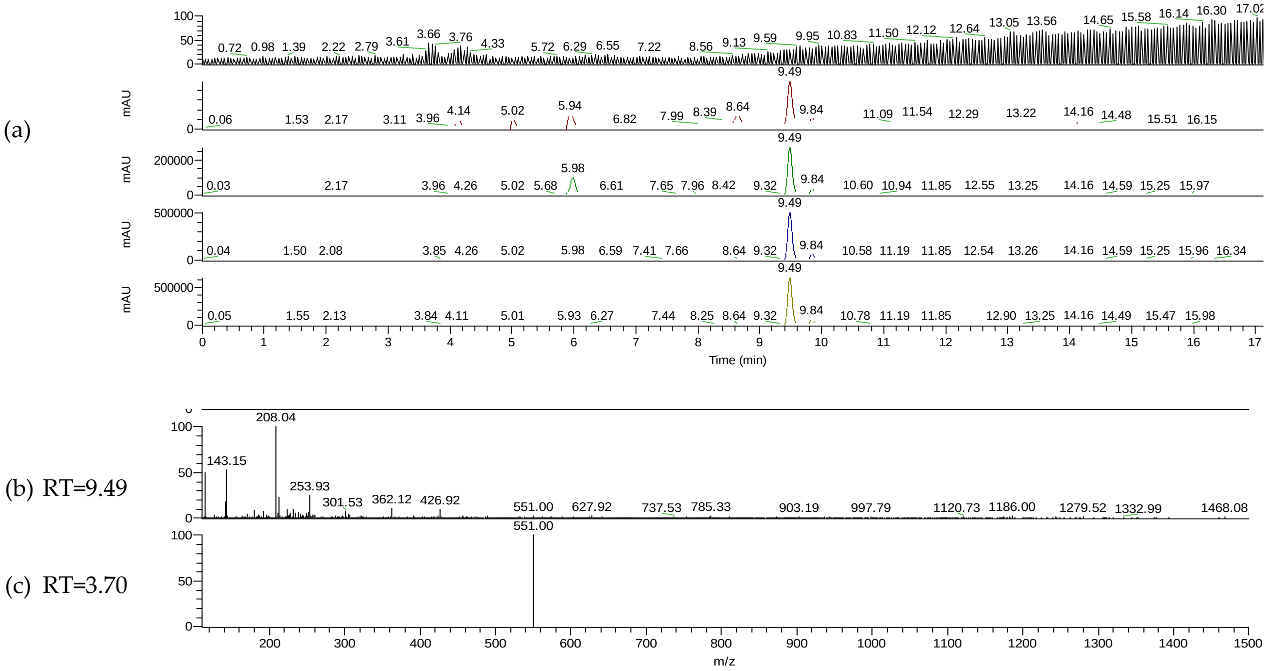

Figure S1. HPLC chromatogram (**a**) and mass spectra (**b** and **c**) of the compounds identified in the Rex1 extract.

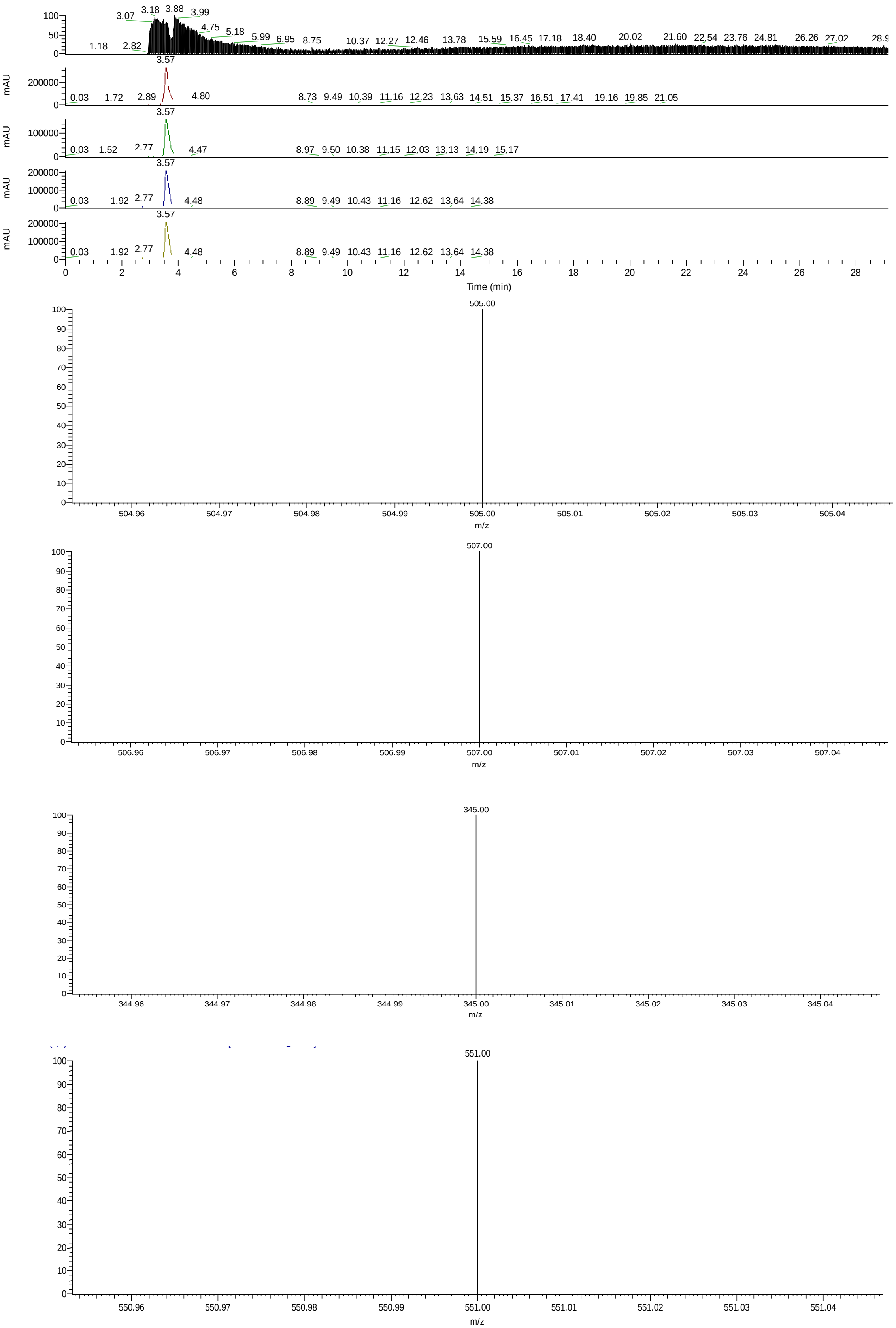

(a)

(b) RT=2.77

(c) RT=3.18

(d) RT=3.88

(e) RT=3.89

Figure S2. HPLC chromatogram (**a**) and mass spectra (**b**-**e**) of the compounds identified in the PresEx1 extract.
